# The Rationale of the Administration of Mercurials

**Published:** 1861-02

**Authors:** John C. K. Crooks

**Affiliations:** Middleburg, Va.


### THE RATIONALE OF THE ADMINISTRATION OF MERCURIALS.

By John C. K. Crooks, M. D., of Middleburg, Va.

The precise method in which mercurials act as remedial agents, has, until quite recently, been comparatively misunderstood. Writers upon materia medica, therapeutics, etc., have widely differed in their notions; while practitioners of medicine have administered this valuable class of remedies with indications often in direct antagonism. Cullen sets down the action of mercurials as "stimulant," "diuretic," "aperient," and "deobstruent." Armstrong, as medicines "equalizing the circulation." Hooper, as having "two effects:" "one as a stimulus cn the constitution and particular parts; the other, as a specific on a diseased action of the whole body, or of parts;" adding, as an evidence of the ignorance of its precise effects, that "the latter action can only be computed by the *disease disappearing.*" Eberle, as "producing a new disease," "equalizing the circulation,"

"revulsive," "evacuant," etc. Wood and Bache, as "purgative," "alterative," "sialagogue," and "sedative." While Drs. Murray and Chapman, approaching more nearly to a correct perception of their true operation, class them among the *tonics*.

This multiplicity of testimony, even from authors of a recent day, it will be discovered, comes wide of a *rational* explanation of the *modus operandi* of those remedies, which have gained such a deservedly exalted reputation as reliable combatants of disease. Yet notwithstanding this evidence of what mercurials *can do*—this record of *effects*, they have all along been operating upon the human organism, in health and disease, with unerring uniformity. With the intelligent practitioner, some form of mercury has come to be considered the sheet-anchor in acute inflammations, particularly of serous and fibrous tissues. Popular prejudice *will not do* with the man who has the well-being of his patient at heart, and properly appreciates the value of this great *anti*-phlogistic remedy. When "this trembling house of clay" is invaded by such a grave disease as an acute inflammation of one of the vital organs, blood-letting, antimony, opium, etc., may come in for their share of the triumph; but the king who can *rule* the conflict, meet the indication *best*, is mercury. This, experience has taught us to be the case. Intelligently administered—given at the proper time and under proper circumstances—aided when aid is necessary, it never disappoints.

This being true, it is my object to point out the *rationale* of a class of remedies so invaluable, and yet so destructive, under certain circumstances, to those organs which it is the aim of the conservative dentist to preserve. In doing so I shall not claim to say anything *new*, (for I believe the field has been pretty well explored before me,) yet a new *version* of the story may not be entirely unacceptable.

To make my subject clearly understood, I must first be allowed to consider briefly the two pathological conditions—congestion and inflammation. In performing this task, we must bear in mind the circulatory system, and with it the constituent elements of the blood—its rough anatomy, rather than its chemistry. In the investigations of microscopists, it has been ascertained that there are two "stages" in every case of active congestion: the stage of "incubation," and the stage of "actual development;" while in inflammation there are three: the stage of "incubation," "active congestion," and lastly, (as in active congestion,) the stage of "actual development," or that pathological condition *known as inflammation.*

To illustrate: let the web of a frog's foot be wounded while under the field of the microscope, and as the first result, there will follow an irregular circulation of the blood in the capillaries, which is neither congestion nor inflammation, but the *immediate* effects of the injury: probably upon the nerves of the part, and so through them upon the capillary vessels. This over—this *irregularity*—and there follows a short period in which every thing goes on in a normal manner, without any evidence of the violence done to the parts. This is termed the period of "incubation." Then comes "active congestion," evinced by an increased afflux of blood with an acceleration of its velocity,—by the distention and *tensity* of the vessels, the interruption of the secretion and absorption of lymph, the apparent agglutination of the blood-globules into masses, and tumefaction of the parenchyma of the web. This condition of things goes on increasing till inflammation is developed by *the loss of capillary power*, (contractility,) and the consequent dilation of these vessels, and from dilation the establishment of "*points of stagnation*" in the current of blood. Here, then, we get the morbid changes upon which active congestion and inflammation depend; the former consisting in an *increased flow of blood to the part*, with the consequences of such determination as already enumerated; while the latter consists in the *dilatation of the capillaries from loss of capillary powers*, and the formation of "*points of stagnation.*"

To the inquiring physician this revelation of the microscope is pregnant with meaning, and if not already correct, must produce a revolution in all his notions of inflammation —cause him effectually to abandon the idea that this pathological state is "increased *activity of the circulation.*" He thus revises his pathology; but, having seen the efficacy of mercurials, he does not throw them away for something imaginary and untried, to meet this new indication. No! But he studies anew and understandingly the manner of their operation, and sees at once that where he formerly gave mercury as a "revulsive," "to equalize the circulation," as a "stimulant," an "evacuant," etc., he now administers it to meet that important indication—the *restoration of the* TONE

*of the capillaries.* Reviewing the series of changes which make up inflammation—the order of sequence—he discovers that the first step to be taken is to restore capillary power. Venesection, he has been taught, will often do it. Antimony, he has learned, will frequently accomplish the end. Yet *experience* has shown him that, after blood has been drawn and antimony given till the *volume* of the circulation is reduced to a fearful extent, still that evidence of recuperation in the strength of the capillaries, so anxiously looked for, does not follow. In other words, the lancet has been used to *empty* the dilated capillaries, that *nature* might take advantage of the circumstances and come to the rescue. But nature is worn out; there has been too great a conflict; more *assistance* must be given, and mercury *completes the work*—completes it when *nothing else will.* If mercury is able to do this, how can we account for its action, save to point at what it *invariably accomplishes*, when it is able to accomplish anything—namely, to RESTORE CAPILLARY POWER?

Such undoubtedly *is* the specific action of mercurials; and, keeping this principle in view, there will be a wonderful clearing up of many of the apparent inconsistencies, incongruities, and mysteries of their operation. Their "alterative" effect in chronic inflammations is immediately revealed. They correct the unhealthy state of the capillaries by causing them to contract upon their contents. As a promoter of absorption it is seen to be by their power to lessen the calibre of the capillaries—the diminishing the flow of blood to a part, and thus literally *starving*, (like tying the carotid artery for tumor of the antrum,) instead of giving energy to the absorbents. This specific effect, as we have seen, is upon the systemic circulation, yet it appears to be more powerful in that portion confined to the salivary glands and the mucous lining of the mouth. Here the *intensity* of the contractility excited is followed by a *loss* of tone, and a consequent relaxation and distension of the vessels—or inflammation; just as in chilblain, where excessive cold produces contraction to such an extent that a loss of capillary power is effected, and inflammation ensues. And here I might speak of the *effects* of cold upon the system when it is under the influence of mercurials, as further evidence in support of the doctrine given. That *cold* causes the capillaries to contract, no one will deny; still such contraction, carried to a moderate extent, as under ordinary circumstances, *except* in an individual *mercurialized*, is productive of no unpleasant consequences. But when mercury has already *produced* contraction, the application of another agent, which has the power to contract the vessel still more, is followed by inflammation in the more susceptible tissues: in the *fibrous*, producing *rheumatism;* in the mouth, (from drinking ice water, for instance,) mercurial stomatitis, etc.

Thus I have endeavored, as briefly as possible, to give the rationale of the operation of mercurials, together with an explanation of some of the phenomena attending their administration. To go into further details would not be profitable in an article of this character. However, to such as may desire to pursue the investigation, I would most earnestly recommend the perusal, ay, *study* of "Billing's Principles of Medicine," a work *replete* with useful knowledge, both to the dentist and the practitioner of medicine.—*Dental Cosmos.*

